# A Practical Handbook on the Muscular Anomalies of the Eye

**Published:** 1899-06

**Authors:** 


A Practical Handbook on the Muscular Anomalies of the Eye. By Howard F. Hansell, A. M., M. D., Clinical Professor of Ophthalmology, Jefferson Medical College; Professor of Diseases of the Eye, Philadelphia Polyclinic and College for Graduates in Medicine; Consulting Ophthalmologist to the Chester County Hospital; Ophthalmologist to Frederick Douglass Memorial Hospital; and Wendell Reber, M. D., Instructor in Ophthalmology, Philadelphia Polyclinic and College for Graduates in Medicine; one of the Ophthalmologists to the Methodist-Episcopal Orphanage. Twenty-eight illustrations and one plate; 182 pages. Philadelphia: P. Blakiston's Son & Co. 1899.

Hansell and Reber have produced a useful and safe guide to the treatment of anomalies of the eye-muscles. This subject is at present engaging the attention of the profession to quite an extent, and its conservative consideration by these authors is timely. After an introductory chapter on the anatomy and physiology of the muscles of the eyes, follows a concise description of their structural and functional anomalies and their treatment, with a concluding section on operations on the muscles. This little work affords good reading, not only for the ophthalmologist, but for the general practitioner as well. A. A. H.

